# Dorsal root ganglion transcriptome analysis following peripheral nerve injury in mice

**DOI:** 10.1177/1744806916629048

**Published:** 2016-03-11

**Authors:** Shaogen Wu, Brianna Marie Lutz, Xuerong Miao, Lingli Liang, Kai Mo, Yun-Juan Chang, Peicheng Du, Patricia Soteropoulos, Bin Tian, Andrew G. Kaufman, Alex Bekker, Yali Hu, Yuan-Xiang Tao

**Affiliations:** 1 Department of Obstetrics and Gynecology, Nanjing Drum Tower Hospital, Nanjing University Medical School, Jiangsu, China; 2 Department of Anesthesiology, New Jersey Medical School, Rutgers, The State University of New Jersey, Newark, NJ, USA; 3 Department of Anesthesiology and Intensive Care, Eastern Hepatobiliary Surgical Hospital, The Second Military Medical University, Shanghai, China; 4 Department of Anesthesiology, Zhujiang Hospital, Southern Medical University, Guangzhou, Guangdong, China; 5 High Performance and Research Computing, Office of Information Technology, New Jersey Medical School, Rutgers, The State University of New Jersey, Newark, NJ, USA; 6 Departments of Biochemistry & Microbiology, New Jersey Medical School, Rutgers, The State University of New Jersey, Newark, NJ, USA; 7 Departments of Cell Biology & Molecular Medicine, New Jersey Medical School, Rutgers, The State University of New Jersey, Newark, NJ, USA; 8 Department of Pharmacology & Physiology and Neuroscience, New Jersey Medical School, Rutgers, The State University of New Jersey, Newark, NJ, USA

**Keywords:** Dorsal root ganglion, neuropathic pain, next generation sequencing, transcriptomes

## Abstract

**Background:**

Peripheral nerve injury leads to changes in gene expression in primary sensory neurons of the injured dorsal root ganglia. These changes are believed to be involved in neuropathic pain genesis. Previously, these changes have been identified using gene microarrays or next generation RNA sequencing with poly-A tail selection, but these approaches cannot provide a more thorough analysis of gene expression alterations after nerve injury.

**Methods:**

The present study chose to eliminate mRNA poly-A tail selection and perform strand-specific next generation RNA sequencing to analyze whole transcriptomes in the injured dorsal root ganglia following spinal nerve ligation. Quantitative real-time reverse transcriptase polymerase chain reaction assay was carried out to verify the changes of some differentially expressed RNAs in the injured dorsal root ganglia after spinal nerve ligation.

**Results:**

Our results showed that more than 50 million (M) paired mapped sequences with strand information were yielded in each group (51.87 M–56.12 M in sham vs. 51.08 M–57.99 M in spinal nerve ligation). Six days after spinal nerve ligation, expression levels of 11,163 out of a total of 27,463 identified genes in the injured dorsal root ganglia significantly changed, of which 52.14% were upregulated and 47.86% downregulated. The largest transcriptional changes were observed in protein-coding genes (91.5%) followed by noncoding RNAs. Within 944 differentially expressed noncoding RNAs, the most significant changes were seen in long interspersed noncoding RNAs followed by antisense RNAs, processed transcripts, and pseudogenes. We observed a notable proportion of reads aligning to intronic regions in both groups (44.0% in sham vs. 49.6% in spinal nerve ligation). Using quantitative real-time polymerase chain reaction, we confirmed consistent differential expression of selected genes including Kcna2, Oprm1 as well as lncRNAs Gm21781 and 4732491K20Rik following spinal nerve ligation.

**Conclusion:**

Our findings suggest that next generation RNA sequencing can be used as a promising approach to analyze the changes of whole transcriptomes in dorsal root ganglia following nerve injury and to possibly identify new targets for prevention and treatment of neuropathic pain.

## Background

Neuropathic pain resulting from nerve injury is a complex and debilitating public health concern. It is characterized by hyperalgesia, allodynia, and spontaneous pain.^1^ Opioids provide effective relief of these symptoms, but extended opioid use produces hyperalgesia and tolerance, eliminating their effectiveness in chronic neuropathic pain relief.^2^ Identifying the mechanisms of peripheral nerve injury-induced neuropathic pain is essential for the discovery of novel treatments and preventative tactics for this disorder. It has been found that peripheral nerve injury produces abnormal ectopic activity at the neuroma of injured sites and within dorsal root ganglia (DRG) neurons.^3,4^ This abnormal activity, which has been identified as a neuropathic pain contributor, may be related to changes in the expression of noncoding RNAs (ncRNAs), receptors, ion channels, and other intracellular proteins in DRG neurons.^5–8^ Previous studies used gene microarrays to identify changes in the RNA transcript expression of several annotated genes within the DRG following peripheral nerve injury,^5,9,10^ but full analysis of the transcriptome and comprehensive gene expression patterns in neuropathic pain is still elusive.

Next-generation RNA sequencing (RNA-seq) is a highly sensitive method of analyzing differential expression of not only mRNAs but also ncRNAs and splice variants. Unlike gene microarrays, RNA-seq is not limited to a subset of known genes for analysis and can map reads to the entire genome of tissues and organs, providing information on possible functions of previously unannotated genes.^11^ Additionally, by neglecting to select for poly-A tailed mRNA, RNA-seq can detect more ncRNAs, as it has been reported that several ncRNAs lack poly-A tails.^12^ However, previous studies carried out next-generation RNA-seq with poly-A tail selection in the DRG under neuropathic pain conditions.^6,13–15^ To this end, the present study chose to perform next-generation RNA-seq with a higher sequencing depth and without mRNA poly-A tail selection to analyze the transcriptome of DRG following spinal nerve ligation (SNL). Compared to previous studies,^6,13–15^ we identified more splice variants as well as more long ncRNAs (lncRNAs) that displayed significant expression changes in DRG after SNL. Our findings provide novel information that can be used to identify key players in neuropathic pain mechanisms.

## Methods

### Animal preparation

Eight-week-old male C57BL/6 mice were obtained from Charles River Laboratories (Raleigh, NC) and were housed in the central housing facility under a standard 12 h light/12 h dark cycle at Rutgers New Jersey Medical School, Newark, NJ. Food and water were provided ad libitum. The Animal Care and Use Committee at Rutgers New Jersey Medical School approved all procedures used in this study. Additionally, all procedures are consistent with ethical guidelines produced by the National Institutes of Health and the International Association for the Study of Pain. Every effort was made to minimize the number of animals used and animal suffering. Experimenters were blinded to the treatment conditions of all animals.

### L4 SNL-induced neuropathic pain model

For RNA-seq, SNL was performed on the forth lumbar (L4) spinal nerve according to our previous methods.^16,17^ As several DRG were required for RNA-seq, we carried out bilateral SNL to minimize the amount of animals used. For qPCR verification experiments, unilateral SNL was performed to provide validation that the RNA-seq results are repeatable. Mice were anesthetized with isoflurane and the L4 spinal nerve was exposed through removal of the L6 transverse process. After exposure and isolation of the L4 spinal nerve, a tight ligation with 7–0 silk thread was made and the nerve was transected distal to the ligature. The surgical procedure for the sham group was identical to that of the SNL group, except that the spinal nerves were not transected or ligated.

### Behavioral testing

Paw withdrawal responses to mechanical stimuli: Mice were placed in individual Plexiglas chambers elevated on a mesh screen. Calibrated von Frey filament (0.38 g) was used to stimulate the center of each hind paw for 1 second. Each application represented one trial, and 10 trials were completed for each hind paw. The time between stimulation of the left paw and stimulation of the right paw was 5 minutes. The number of times the mouse exhibited a response (withdrawal, flicking, and licking) to the filament was recorded for each set of 10 trials, producing a frequency ([number of paw withdrawals/10 trials] × 100 = % response frequency). This frequency was used as a measurement of mechanical allodynia.

### RNA extraction

Bilateral L4 DRG from SNL and sham groups were harvested six days after surgery, immediately treated in RNAlater (Ambion, Austin, TX) and subjected to total RNA extraction. To obtain enough RNA for sequencing, eight L4 DRGs from four mice were pooled together and each group required three biological replicates (*n* = 12 mice/group). For quantitative real-time PCR assay, four L4 DRGs from the ipsilateral sides of four SNL or sham mice were pooled together and the experiment was repeated three times (*n* = 12 mice/group). Briefly, total RNA was extracted using the miRNeasy kit with on-column digestion of genomic DNA (QIAGEN, Valencia, CA) according to the manufacturer’s instructions. RNA concentration was measured using the NanoDrop 2000 Spectrophotometer (Thermo Scientific, Wilmington, DE) and Qubit Fluorometric Quantitation (Invitrogen, Carlsbad, CA). Ratios of A260/280 nm were between 1.97 and 2.08. RNA integrity was assessed using RNA Nano chips in an Agilent 2100 Bionalyzer (Agilent technologies, Santa Clara, CA). RNA integrity numbers (RIN) were between 7.5 and 8.4.

### RNA sequencing

The above total RNA extracted (1.2 µg/sample) was subjected to rRNA depletion by Ribo-Zero rRNA Removal (Human/Mouse/Rat) Kit (Illumina, San Diego, CA). Strand-specific RNA libraries were prepared using TruSeq Stranded Total RNA Sample Preparation Kit (Illumina) without poly-A selection. All assays were performed according to the manufacturer’s instructions. Sequencing was carried out at the Illumina HiSeq2500 platform High Output Mode, in a 2 × 100 base pairs paired-end configuration, with a total of more than 190 M reads per lane (at least 60 M reads per sample).

### Bioinformatics analysis

Six samples were subjected to multiplexing, sequencing, differential gene expression analysis, transcript expression analysis, and ncRNA expression analysis. Briefly, the sequences were quality trimmed using trimmomatic-0.32 (minimal length 50 base pairs, leading and trailing Phred Q 30) first. The resulting sequencing data were then mapped to the musculus genome sequence version GRCm38.72, downloaded from ENSEMBLE. Gene hit counts and reads per kilobase per million (RPKM) mapped reads were calculated for each gene to determine expression levels.^11^ Comparisons of gene expression between groups were conducted using Student’s *t*-test. The long ncRNA was identified according to the annotation of biotype. The above RNA-seq analysis, including mapping, read counts, and expression analysis were performed within the CLCbio software environment (CLC genomics Workbench 7.0.2, CLC genomics Server). Mapped reads were visualized on the UCSC browser using bigwig files converted from bam files.

### Pathway analysis

For biological pathway categorization, a total of 237 genes were selected based on their significance (false discovery rates, *p* < 0.05), differential expression (greater than normalized two-fold change following nerve injury), and ability to be categorized using the Panther Classification System database. Using the PANTHER (© 2015, Paul Thomas) system (pantherdb.org), we grouped genes based on biological pathways.

### Quantitative real-time RT-PCR

Total RNA was reverse transcribed using the ThermoScript reverse transcriptase (Invitrogen) according to the manufacturer’s instructions with either the oligo (dT) primers or specific RT primers. cDNA was amplified by real-time PCR using the primers listed in Table 1 (Integrated DNA Technologies, Coralville, IA). Each sample was run in triplicate in a 20 μl reaction with 250 nM forward and reverse primers and 10 μl of Advanced Universal SYBR Green Supermix (Bio-Rad Laboratories, Hercules, CA). Reactions were performed in a BIO-RAD CFX96 real-time PCR system. The cycle parameters were set as follows: an initial 3-min incubation at 95℃, followed by 40 cycles of 95℃ for 10 s, 60℃ for 30 s, and 72℃ for 30 s. Ratios of ipsilateral-side mRNA levels to contralateral-side mRNA levels were calculated using the ΔCt method (2-ΔΔCt) at a threshold of 0.02. All data were normalized to Tubala (tubulin alpha 1 A gene), which was demonstrated to be stable after SNL in our pilot work.

**Table 1.** Primers for RT-qPCR.

Primer names	Sequences
Tuba1a–RT	TTC CAC AGA ATC CAC ACC AA
Kcna2-RT	GTC CCC GTC ACA TCT TCT CAC T
Gm21781-RT	CGG TGA TAC CAG CAC ACA TC
4732491K20Rik-RT	CAG CAT CCA TGT GAA CCA AC
Tuba1a–F	GTG CAT CTC CAT CCA TGT TG
Tuba1a–R	GTG GGT TCC AGG TCT ACG AA
Kcna2-F	CTG CAA GGG CAA CGT CAC AC
Kcna2-R	GGG ACA GTG AGA TGC TTG GC
Oprm1-F	TCT TCA CCC TCT GCA CCA TG
Oprm1-R	TCT ATG GAC CCC TGC CTG TA
Gm21781-F	GAC CTT TGG AAG AGC AGT CG
Gm21781-R	CAG CCT GGT CTA CAC AGC AA
4732491K20Rik-F	GGC CAA AAG ATT CCT CCT TC
4732491K20Rik-R	TAG TGC CAG GCT GAC CTT CT

RT-qPCR: reverse transcriptase quantitative polymerase chain reaction; F: Forward; R: Reverse.

### Statistical analysis

All data were collected randomly and expressed as mean ± SEM. The data were statistically analyzed with two-way analysis of variance (ANOVA) or paired Student’s *t* test. When ANOVA showed a significant difference, pairwise comparison between means were tested by the post hoc Turkey method. *p* values less than 0.05 were considered statistically significant.

## Results

### Development of SNL-induced mechanical allodynia in mice

**Figure 1.**
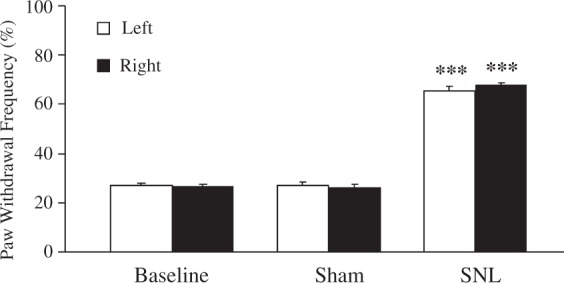
Bilateral L4 spinal nerve ligation produced mechanical allodynia in mice. SNL mice (*n* = 12) had significantly increased paw withdrawal frequencies in response to a .38 g von Frey hair for both hind paws six days after surgery compared with the sham group (*n* = 12). The results represent the mean ± SEM; **p < 0.01 compared with the sham group. SNL: spinal nerve ligation.

To make sure that all SNL mice used in the sequencing experiments showed pain hypersensitivity, mouse paw withdrawal frequency in response to mechanical stimulation was carried out. Consistent with our previous studies,^16^ SNL led to mechanical allodynia as demonstrated by a dramatic increase in paw withdrawal frequency for both hind paws on Day 6 post surgery (a time point at which altered gene expression and associated pain-related behavior are well established),^16,17^ compared to baseline (Figure 1). As expected, sham surgery did not produce a change in paw withdrawal frequency in either hind paw (Figure 1).

### RNA-seq and genome-wide read mapping in DRGs after nerve injury

Through the use of illumine paired end sequence, 100 base pair long reads were yielded. More than 50 million (M) reads were identified per sample (51.87 M–56.12 M in the sham surgery group vs. 51.08 M–57.99 M in the SNL group; *p* = 0.68). Reads filtered using Illumina quality control were mapped to the reference mouse genome from ENSEMBLE (GRCm38.72), permitting up to one mismatch when aligning to the genome. The filtered reads that did not map to the genome or that could map to more than one genomic location were excluded from the analysis.

**Figure 2.**
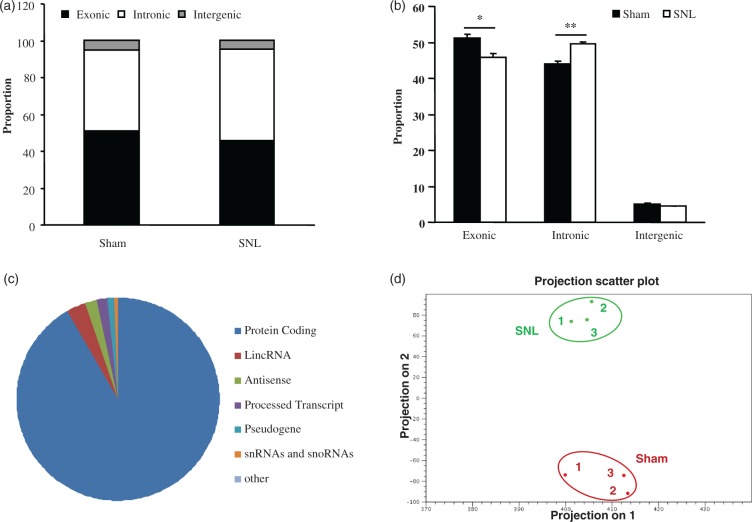
Transcriptome profiling in mice fourth lumbar dorsal root ganglia after nerve injury. (a) The mapped proportions of exonic, intronic, and intergenic reads in sham and SNL group. (b) There is a significantly lower proportion of reads that align to exonic regions in SNL samples when compared with sham samples, and the proportion of reads that align to intronic regions is significantly higher in SNL samples compared with sham samples. (c) The distribution of differentially expressed RNAs in the fourth lumbar dorsal root ganglia associated with peripheral nerve injury. (d) PCA was performed using the log2-transformed and quantile-normalized RPKM values. The colors indicate different groups of samples. The results represent the mean ± SEM of three independent experiments. *p < 0.05; **p < 0.01 compared with the sham group. PCA: Principle component analysis; RPKM: reads per kilobase per million; SNL: spinal nerve ligation.

Mapped reads were categorized as exonic, intronic, or intergenic. Figure 2(a) illustrated the proportion of the reads belonging to each category in the sham and SNL groups. As expected, many reads were aligned to exonic regions in both groups (Figure 2(a)). Interestingly, a high proportion of reads also mapped to intronic regions in the sham and SNL groups (44.0% in the sham group and 49.6% in the SNL group; Figure 2(a)). In contrast, only 4.9% and 4.5% of reads mapped to intergenic regions in the sham and SNL groups, respectively (Figure 2(a)). Further analysis showed that SNL significantly decreased the proportion of reads aligning to exonic regions (*p* < 0.05), increased the proportion of reads aligning to intronic regions (*p* < 0.01), and did not significantly alter the proportion of reads aligning to intergenic regions, when compared with the sham group (Figure 2(b)).

Finally, differentially expressed RNAs in the DRG associated with peripheral nerve injury were detected. Six days after SNL, the expression levels of 11,163 genes out of a total of 27,463 identified genes were significantly altered in the L4 DRG of SNL mice compared with the sham group (*p* < 0.05), of which 52.14% were upregulated and 47.86% downregulated. As expected, the largest transcriptional changes were seen in protein-coding RNAs (91.5%) (Figure 2(c)). Of the remaining differentially expressed RNAs, the largest transcriptional changes were observed in long interspersed ncRNAs (lincRNAs) followed in order by antisense RNAs, processed transcripts, pseudogene ncRNAs, snRNAs and snoRNAs, and others (e.g., nonsense-mediated decay) (Figure 2(c)). The variation of these differentially expressed RNAs across the samples from the sham and SNL groups was examined via principal component analysis. This analysis separated the samples into two clusters corresponding to the sham and SNL groups, respectively (Figure 2(d)), indicating that the robust differences in the changes of RNA expression occurred between the sham and SNL groups and not within groups.

### Highest differentially expressed RNAs in the injured DRG following peripheral nerve injury

**Figure 3.**
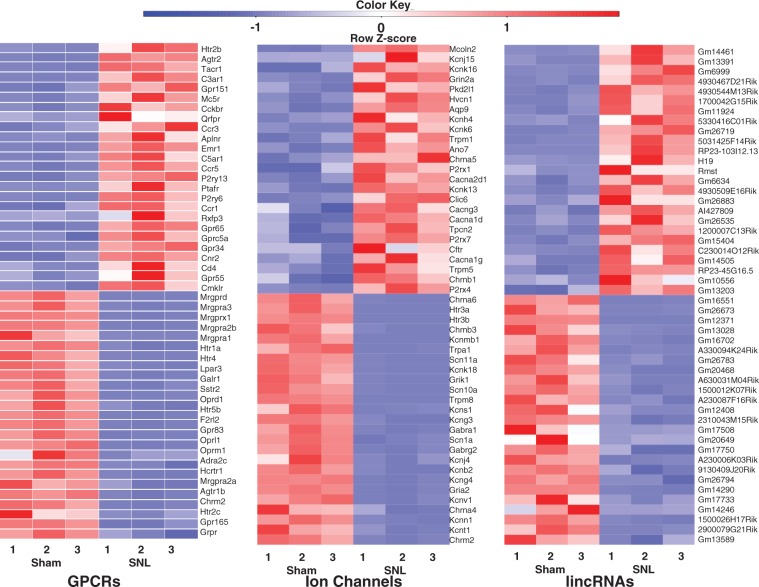
Representative differentially expressed genes in fourth lumbar dorsal root ganglia after nerve injury. The 25 most up- and downregulated G protein-coupled receptors (a), ion channels (b), and long intergenic noncoding RNAs (c) in the fourth lumbar dorsal root ganglia are represented in the form of scaled heat maps created using *Z*-score values obtained using RNA-seq. RNA-seq: RNA sequencing.

**Figure 4.**
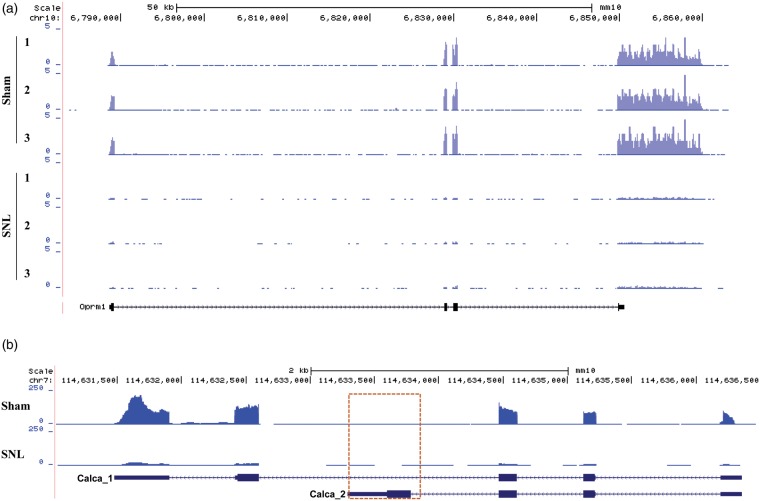
Visualized reads mapping to the genomic location of differentially expressed genes and variants. (a) The stacked reads decreased in SNL samples significantly compared with sham samples in the genomic region of the MOR gene. (b) Alternative splicing analysis identified variant *Calcal* of the calcitonin/calcitonin gene-related polypeptide, alpha gene. The expression of *Calca_2,* the variant encoding calcitonin, was too low to be detected in DRG. The open box indicates that few reads were aligned to the unique exon region of *Calca_2*. SNL: spinal nerve ligation; MOR: mu opioid receptor; DRG: dorsal root ganglia.

**Figure 5.**
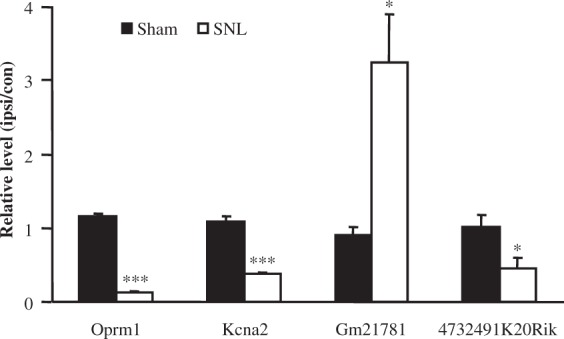
Validation of differentially expressed genes by qPCR. qPCR validated that Gm21781 RNA is significantly upregulated after SNL and Oprm1mRNA, Kcna2 mRNA, and 4732491K20Rik RNA are downregulated. The results represent the mean ± SEM of three independent experiments. *p < 0.05; **p < 0.01 compared with the sham group. qPCR: quantitative polymerase chain reaction; SNL: spinal nerve ligation.

G protein-coupled receptors (GPCRs), ion channels, and lncRNAs play a key role in regulating the transmission of peripheral nociceptive information.^18–23^ Table 2 depicted expression changes in some of these identified genes that are highly relevant to pain, itch, touch, thermal, and chemical sensitivity. We first analyzed the top 25 significantly (*p* < 0.05) upregulated and top 25 significantly (*p* < 0.05) downregulated GPCR genes from the identified 403 GPCR genes in the injured DRG six days after SNL (Figure 3(a); Table 2). Consistent with previous studies, the type-2 angiotensin II receptor,^24^ substance P receptor,^25^ and C-C chemokine receptor type 5^26^ increased after nerve injury. Additionally, the RNA-seq exhibited decreases in the mu-type opioid receptor,^27^ somatostatin receptor type 2,^28^ and Galanin receptor type 1,^29^ which was consistent with previous studies. All of the aforementioned GPCRS have known functions in neuropathic pain. The top 25 significantly (*p* < 0.05) upregulated and top 25 significantly (*p* < 0.05) downregulated ion channel genes (out of a total of 303 RNA-seq identified ion channels) are depicted in the heat map in Figure 3(b) and Table 2. Interestingly, RNA-seq data showed that genes Kcnk13,^30^ Chrna5,^31^ and Cacna2d1^32^ increased following nerve injury, which is consistent with previous studies that found the functional significance of these genes in pain. Also consistent with previous studies, Scn10a, Scn11a,^33^ Gabra1,^34^ and Kcns1^35^ decreased after SNL in our RNA-seq results. Our sequencing methods allowed for the identification of more lincRNAs than previous studies, as we neglected to perform poly-A tail selection. The top 25 significantly (*p* < 0.05) upregulated and top 25 significantly (*p* < 0.05) downregulated lincRNAs (out of a total of 1,597 RNA-seq identified lincRNAs) were displayed in the heatmap in Figure 3(c). As the discovery of lincRNAs is still at its infancy, the RNA-seq expression analysis of the lincRNAs depicted in Figure 3(c) is novel. Figure 4(a) depicts a representative example of RNA-seq reads for a differentially expressed gene. In this example, the reads mapped to the genomic location corresponding to Oprm1, the mu opioid receptor (MOR) 1 gene, for all samples in each group. MOR has previously been found to be downregulated in the injured DRG after peripheral nerve injury.^36^ In Figure 4(a), the stacked reads significantly decreased in the SNL samples compared with the sham samples. The reliability in RNA-seq was demonstrated by the repeatable decrease in MOR in all three SNL samples. Our further validation experiments using quantitative real-time reverse transcription polymerase chain reaction (qPCR) analysis confirmed consistent differential expression of selected genes including Kcna2, Oprm1 as well as lncRNAs Gm21781 and 4732491K20Rik (Figure 5).

**Table 2.** Changes in some identified genes that are highly relevant to pain, itch, touch, thermal, and chemical sensitivity in the injured DRG after SNL.

Upregulation			Downregulation		
Name	Description	Ratio change	Name	Description	Ratio change
Agtr2	Angiotensin II receptor, type 2	25.34	Adra2c	Adrenergic receptor, alpha 2 c	0.09
Aqp9	Aquaporin 9	4.35	Asic3	Acid-sensing (proton-gated) ion channel 3	0.39
Atf3	Activating transcription factor 3	9.64	Atp1a1	ATPase, Na+/K+ transporting, alpha 1 polypeptide	0.27
Cacna1d	Calcium channel, voltage-dependent, L type, alpha 1D subunit	2.30	Atp1b3	ATPase, Na+/K+ transporting, beta 3 polypeptide	0.63
Cacna1g	Calcium channel, voltage-dependent, T type, alpha 1 G subunit	2.18	Calca	Calcitonin/calcitonin-related polypeptide, alpha	0.14
Cacna1h	Calcium channel, voltage-dependent, T type, alpha 1 H subunit	1.05 (n.s.)	Chrna4	Cholinergic receptor, nicotinic, alpha polypeptide 4	0.12
Cacna2d1	Calcium channel, voltage-dependent, alpha2/delta subunit 1	3.07	Chrna6	Cholinergic receptor, nicotinic, alpha polypeptide 6	0.01
Cacng3	Calcium channel, voltage-dependent, gamma subunit 3	2.31	Chrnb3	Cholinergic receptor, nicotinic, beta polypeptide 3	0.02
Cckbr	Cholecystokinin B receptor	10.00	Gabbr1	Gamma-aminobutyric acid (GABA) B receptor, 1	0.40
Ccr1	Chemokine (C-C motif) receptor 1	5.67	Gabra1	Gamma-aminobutyric acid (GABA) A receptor, alpha 1	0.07
Ccr3	Chemokine (C-C motif) receptor 3	8.25	Gabrg2	Gamma-aminobutyric acid (GABA) A receptor, subunit gamma 2	0.10
Ccr5	Chemokine (C-C motif) receptor 5	7.05	Galr1	Galanin receptor 1	0.05
Chrna5	Cholinergic receptor, nicotinic, alpha polypeptide 5	3.25	Gria2	Glutamate receptor, ionotropic, AMPA2 (alpha 2)	0.11
Gal	Galanin	10.06	Grik1	Glutamate receptor, ionotropic, kainate 1	0.05
Grin2a	Glutamate receptor, ionotropic, *N*-methyl D-aspartate 2 A	6.64	Htr1a	5-Hydroxytryptamine (serotonin) receptor 1 A	0.04
Htr2b	5-Hydroxytryptamine (serotonin) receptor 2B	35.49	Htr2c	5-Hydroxytryptamine (serotonin) receptor 2 C	0.14
Hvcn1	Hydrogen voltage-gated channel 1	5.90	Htr3a	5-Hydroxytryptamine (serotonin) receptor 3a	0.01
Kcnh4	Potassium voltage-gated channel, subfamily H (eag-related), member 4	4.02	Htr3b	5-Hydroxytryptamine (serotonin) receptor 3b	0.02
Kcnj15	Potassium inwardly rectifying channel, subfamily J, member 15	7.43	Htr4	5-Hydroxytryptamine (serotonin) receptor 4	0.04
Kcnk13	Potassium channel, subfamily K, member 13	2.74	Htr5b	5-Hydroxytryptamine (serotonin) receptor 5b	0.08
Kcnk16	Potassium channel, subfamily K, member 16	6.80	Htr7	5-Hydroxytryptamine (serotonin) receptor 7	0.44
Kcnk6	Potassium inwardly rectifying channel, subfamily K, member 6	3.84	Kcnb2	Potassium voltage gated channel, Sham-related subfamily, member 2	0.10
Mcoln2	Mucolipin 2	9.84	Kcnc2	Potassium voltage gated channel, Sham-related subfamily, member 2	0.78 (n.s.)
P2rx1	Purinergic receptor P2X, ligand-gated ion channel, 1	3.13	Kcng3	Potassium voltage-gated channel, subfamily G, member 3	0.06
P2rx4	Purinergic receptor P2X, ligand-gated ion channel 4	1.97	Kcng4	Potassium voltage-gated channel, subfamily G, member 4	0.11
P2rx7	Purinergic receptor P2X, ligand-gated ion channel, 7	2.26	Kcnj4	Potassium inwardly rectifying channel, subfamily J, member 4	0.10
P2ry13	Purinergic receptor P2Y, G-protein coupled 13	6.52	Kcnk18	Potassium channel, subfamily K, member 18	0.04
P2ry6	Pyrimidinergic receptor P2Y, G-protein coupled, 6	6.07	Kcnmb1	Potassium large conductance calcium-activated channel, subfamily M, beta member 1	0.03
Pkd2l1	Polycystic kidney disease 2-like 1	6.26	Kcns1	Potassium voltage-gated channel, subfamily S, 1	0.06
Tacr1	Tachykinin receptor 1	15.43	Kcnt1	Potassium channel, subfamily T, member 1	0.13
Tpcn2	Two pore segment channel 2	2.28	Kcnv1	Potassium channel, subfamily V, member 1	0.11
Trpm1	Transient receptor potential cation channel, subfamily M, member 1	3.43	Mrgpra1	MAS-related G protein-coupled receptors, member A1	0.03
Trpm5	Transient receptor potential cation channel, subfamily M, member 5	2.05	Mrgpra2a	MAS-related G protein-coupled receptors, member A2A	0.12
Trpv4	Transient receptor potential cation channel, subfamily V, member 4	1.89	Mrgpra2b	MAS-related G protein-coupled receptors, member A2B	0.03
			Mrgpra3	MAS-related G protein-coupled receptors, member A3	0.01
			Mrgprd	MAS-related G protein-coupled receptors, member D	0.01
			Mrgprx1	MAS-related G protein-coupled receptors, member X1	0.02
			Oprd1	Opioid receptor, delta 1	0.08
			Oprk1	Opioid receptor, kappa 1	0.02
			Oprl1	Opioid receptor-like 1	0.09
			Oprm1	Opioid receptor, mu 1	0.09
			P2ry1	Purinergic receptor P2Y, G-protein coupled 1	0.32
			Piezo2	Piezo-type mechanosensitive ion channel component 2	0.71
			Scn10a	Sodium channel, voltage-gated, type X, alpha	0.05
			Scn11a	Sodium channel, voltage-gated, type XI, alpha	0.04
			Scn1a	Sodium channel, voltage gated, type I alpha subunit	0.09
			Sst	Somatostatin	0.02
			Sstr2	Somatostatin receptor 2	0.05
			Trpa1	Transient receptor potential cation channel, subfamily A, member 1	0.04
			Trpm8	Transient receptor potential cation channel, subfamily M, member 8	0.06
			Trpv1	Transient receptor potential cation channel, subfamily V, member 1	0.33
			Vip	Vasoactive intestinal polypeptide	0.70 (n.s.)

SNL: spinal nerve ligation; DRG: dorsal root ganglia. All ratio changes are significant *p* < 0.05 except if indicated by “n.s.”—no significant.

### Alternative splicing variation after SNL

Our alternative splicing analysis identified a large number of specific differentially expressed variants. For example, calcitonin/calcitonin-related polypeptide alpha (Calca) expression levels decreased (−7.14 fold change of original values, *p* < 0.01) in the SNL group compared with the sham group. Alternative processing of the RNA transcribed from the Calca gene results in the production of calcitonin and another neuropeptide referred to as calcitonin gene-related peptide (CGRP).^37^ Figure 4(b) depicted the reads for each group for two variants of Calca mRNA. RNA-seq revealed a significant decrease (−6.96 fold, *p* < 0.01) in variant Calca_1 (ENSMUST00000032906) but not Calca_2 (ENSMUST00000032907) in the SNL group compared with the sham group. Calca_1 encodes the longer mRNA translating to CGRP1, while Calca_2 encodes the shorter mRNA translating to calcitonin polypeptide.^37^ The expression of Calca_2 is very low in DRG as indicated by the lack of reads in Figure 4(b). This RNA-seq validated decrease in CGRP mRNA in the injured DRG is consistent with previous neuropathic pain studies^38,39^ and further confirms the reliability of next-generation RNA-seq.

### Genes involved in multiple pathways changed following nerve injury

**Figure 6.**
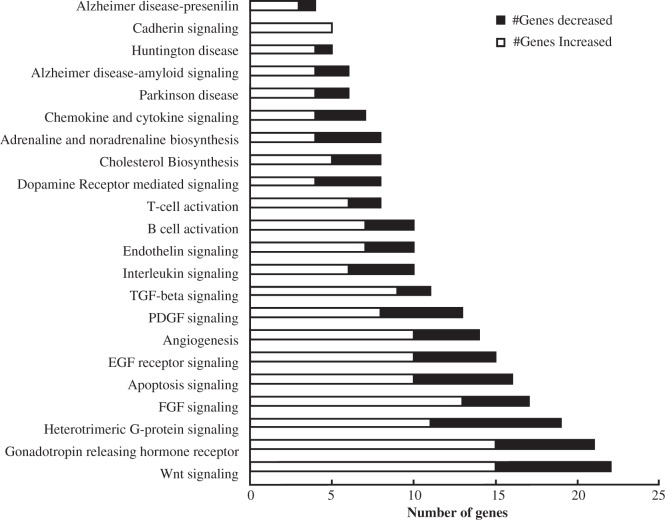
Pathway analyses of the most significant and differentially expressed genes after nerve injury. PANTHER GO analyses groups the most significantly increased (white bars) and decreased (black bars) gene transcripts based on their annotated roles in biological pathways. A total of 237 genes are represented in this pathway analyses, all with greater than a twofold change after nerve injury and a FDR. *p* < 0.05. FDR: false discovery rate.

As our RNA-seq analysis resulted in a substantial amount of differentially expressed genes, we grouped genes based on their participation in biological pathways. Genes were selected for gene ontology pathway analysis after meeting the following criteria: the gene (a) has a greater than twofold change in expression (either increased or decreased) following nerve injury, (b) has an FDR *p* value less than 0.05, and (c) is a coding gene that can be categorized using the PANTHER pathway analysis database. The use of the FDR *p* value in this analysis further ensures that only the most significantly altered genes are included in the pathway analysis. In total, 237 genes met these criteria and were grouped into pathway categories (Figure 6). Notably, the Wnt signaling pathway, G-protein signaling, and Gonadotropin releasing hormone (GnRH) pathways represented major categories as more than 15 genes in each of these pathways were differentially expressed following nerve injury. Previous studies have identified Wnt Signaling as an important pathway involved in neuropathic pain.^40,41^ Interestingly, the expression of most of the genes in these pathways increased rather than decreased after nerve injury. This is consistent with previous gene profiling studies which also found more genes to be upregulated rather than downregulated after nerve injury.^42,43^

## Discussion

Nerve injury is associated with gene expression changes within the DRG. Identifying which genes change and even more importantly how they change is a key factor in defining the mechanism of neuropathic pain development and maintenance. RNA-seq assay reveals in-depth and thorough analyses of the transcriptome changes associated with peripheral nerve injury.

Previous studies carrying out RNA-seq after nerve injury used poly-A tail selection and lower sequencing depths, limiting the amount of ncRNAs and differential genes that can be identified.^6,15^ In the present study, we carried out RNA-seq with no poly-A tail selection and sought to analyze changes in the transcriptome after nerve injury at a greater depth. Additionally, the use of a 100-base pair paired end configuration provides more accurate identification of lncRNAs. However, micro RNAs (miRNAs), due to their size, were overlooked using this configuration. A previous study has focused on identifying miRNAs that change following nerve injury,^6^ therefore, our focus was on lincRNAs. Strand specificity was maintained using the above kit, which is an important tactic for identifying strand-specific orientation of novel genes and ncRNAs (e.g., antisense ncRNA). Our study provides valuable information on differential gene expression, alternative splicing variation, and ncRNA differential expression after peripheral nerve injury.

Differential gene expression analysis using RNA-seq provided information on not only a select group of genes, as is the case with RNA microarrays,^44^ but rather the entire mouse transcriptome. This thorough exploration allows for the discovery of new possible pain targets. Figure 4 provides examples of genes that may have a yet unidentified function in pain, as only a few of the top 25 upregulated and downregulated genes have scientifically validated pain-related functions. Further studies into possible pain-related functions of these differentially expressed GPCRS and ion channels could build on our knowledge of pain mechanisms and possible targets for prevention and treatment of neuropathic pain.

In addition to changes in the expression of known genes, ncRNA differential expression could reveal novel epigenetic mechanisms of pain. ncRNAs include both small ncRNAs, such as miRNA, and lncRNAs. While miRNAs involved in pain have been extensively studied,^6,45^ the roles of lncRNAs in pain are still elusive. LncRNAs include antisense lncRNAs, doubled stranded RNA, and other long RNA species.^46^ We previously reported an antisense lncRNA, Kcna2 antisense RNA, which contributed to neuropathic pain by specific silencing of Kcna2 (or Kv1.2) expression in DRG.^18^ However, with the overwhelming amount of lncRNAs identified in the transcriptome, we anticipate that many more lncRNAs with a pain-related function exist. Many of the identified lincRNAs found in Figure 3(c) have no documented function. As these lincRNAs are differentially expressed after peripheral nerve injury, they could be involved in pain mechanisms in some manner. Validation of the expression of a select number of these unannotated ncRNAs reveals true expression and differential expression in the DRG of mice both before and after nerve injury (Figure 5). Further studies into the functions of these lincRNAs could unveil novel neuropathic pain contributors.

The added benefit of using RNA-seq is the ability to identify differential splicing variation. Alternative splicing is the process in which a single gene can result in multiple mRNAs simply due to exon inclusion/exclusion.^47^ Changes in alternative splicing after nerve injury have been reported^12^ and alternative splicing variation is thought to contribute to drug efficacy variance in chronic pain patients.^48^ In our study, we were able to identify a change in the splicing variation of *Calca,* the gene that encodes for calcitonin or CGRP. RNA-seq showed a decrease in the transcript that translates into CGRP. Interestingly, the *Calcal* transcript decreased −6.96 fold in relation to the sham group (Figure 4(b)). This is very consistent with the reported −7.14 fold change for the entire *Calca* gene. This consistency shows that the *Calca* decrease after SNL may be entirely due to a decrease in the *Calca l* transcript rather than the *Calca_2* transcript. This is expected as calcitonin, the protein translated from the *Calca_2* transcript is predominately expressed in the thymus and CGRP and the protein translated from the *Calca l* transcript is expressed in the DRG.^37^ The low expression of calcitonin in the DRG could explain the lack of a significant change in *Calca_2* transcript expression after SNL. Further analysis of alternative splicing variations after nerve injury could provide insight into how certain genes change in expression in neuropathic pain.

In our study, thousands of genes changed in expression after nerve injury, so grouping these genes based on biological pathways provided an overall view of what may be occurring within the DRG. Our pathway analysis showed that genes involved in GnRH signaling were significantly increased following nerve injury. As males were used for this study, the increase in GnRH signaling would result in testosterone increases in the models. In previous studies of temporomandibular joint (TMJ) pain, gonadal hormones were found to reduce TMJ kappa-mediated antinociception by downregulating the expression of kappa opioid receptors on trigeminal ganglia sensory neurons.^49^ Also, several previous studies have found the expression of kappa opioid receptors in spinal cord and DRG to vary with the estrous cycle.^50–52^ An increase in GnRH following nerve injury may lead to reduced kappa opioid expression in DRG which could lead to pain. This evidence of increased GnRH after nerve injury at the transcription level may provide further validation of the role of sex hormones in neuropathic pain.

## Conclusion

Much of pain-related research to date has focused on select genes that change after nerve injury, but as novel pain treatments are not readily implemented, it is clear that looking beyond the scope of a select subset of genes is necessary. With this study, we identified change at the transcriptome level of not only coding RNAs but also lncRNAs. Our unique and thorough RNA-seq approach provides insight into alternative splicing, which was overlooked by previous RNA-seq and peripheral nerve injury studies. The RNA-seq data obtained from this study have proven to be repeatable as made evident by previous studies and our own qPCR validation experiments. It is our hope that observations made from this article are pursued further with the goal of identifying novel targets for the prevention and treatment of neuropathic pain.
